# Industry Payments to Foot and Ankle-Trained Orthopedic Surgeons: An Analysis of the Open Payments Database From 2014 to 2019

**DOI:** 10.7759/cureus.55354

**Published:** 2024-03-01

**Authors:** John M Tarazi, Nicholas Frane, Alain E Sherman, Josh Giordano, Emma K Humphrey, Peter B White, Adam Bitterman

**Affiliations:** 1 Department of Orthopaedic Surgery, Donald and Barbara Zucker School of Medicine at Hofstra/Northwell, Hempstead, USA; 2 Department of Orthopaedic Surgery, Northwell Health - Huntington Hospital, Huntington, USA; 3 Department of Orthopaedic Surgery, The Center for Orthopedic Research and Education (CORE) Institute, Phoenix, USA; 4 Department of Orthopaedic Surgery, Northwell Health - Lenox Hill Hospital, New York, USA; 5 Orthopedics, Ohio University Heritage College of Osteopathic Medicine, Cleveland, Warrensville Heights, USA

**Keywords:** foot and ankle reconstruction, open payments database, industry payment, orthopaedic surgery, foot and ankle surgeon

## Abstract

Introduction

The authors examined if the transparency in industry payments to foot and ankle-trained orthopedic surgeons resulted in the following changes to the (1) median general payments to surgeons, (2) trend in median payments to surgeons across all subcategory payments, and (3) trend in median payments to surgeons in 11 regions of the United States.

Methods

A retrospective review of the Centers for Medicare and Medicaid Services (CMS) and Open Payments Database (OPD) was performed to identify all industry payments made by drug and medical device companies to orthopedic surgeons (N = 3,835) between January 1, 2014, and December 31, 2019. Descriptive statistics were calculated, and trend analyses in annual payments, number of payments to surgeons per year, payment subtypes, and regional distributions were analyzed.

Results

A total of 53,280 payments totaling $53,454,850.56 were made to orthopedic foot and ankle surgeons between 2014 and 2019. Mean and median payments were $1,003.28 and $60.19, respectively. Statistically significant differences in mean payment amounts were observed by year (p = 0.001) with a highly statistically significant, strong increase in the number of payments made over the six-year period (r = 0.97, p < 0.001). The greatest increases in median individual payments were observed for gifts (277.1%; r = 0.18, p = 0.05), education (250.6%; r = 0.17, p < 0.001), and royalties and licensing (72.1%; r = 0.05, p = 0.04). Statistically significant increasing trends in median payments over time were observed for the Northeast (p < 0.001) and South regions (p < 0.001).

Discussion

The results of this study demonstrate the increase in payments made across the six-year time period. The study demonstrates that there is a shift in the type of payments from speaker fees, entertainment, and lodging to education, gifts, honoraria, royalties, and consulting.

Conclusion

Since the OPD release, no significant decrease was identified in the financial relationship between foot and ankle surgeons and the industry; rather, an increase was observed. This increase in education, royalties, and consulting shows that more foot and ankle surgeons are getting involved in the industry, contrary to expectations. The partnership between industry and physicians can help to improve innovation and bring new ideas to the future of orthopedics.

## Introduction

There have been growing concerns regarding the relationship between physicians and the medical industry. As these relationships continue to allow for the exchange of information and collaboration on innovation, there is a concern that the medical industry can influence physician decisions [1,2]. Furthermore, as financial agreements between physicians and industry continue to grow, conflict of interest disclosure serves as a key step in uncovering the ethical implications of the practice of medicine [3]. To increase transparency, lawmakers implemented the Physician Payments Sunshine Act (PPSA) as part of the United States Patient Protection and Affordable Care Act in 2010 to mandate drug, device, biological, and medical supply manufacturers to report any monetary transfer greater than $10 (US Dollars) to physicians to the Centers for Medicare and Medicaid Services (CMS) [4]. Consequently, CMS founded the National Physician Payment Transparency Program, which developed a publicly available resource for disclosing financial exchanges between physicians and industry known as the Open Payments Database (OPD) [5,6]. Although the OPD was initially met with controversy and viewed as an attempt to deter physicians from accepting payments, others regarded this as an opportunity to strengthen trust within the medical profession [6].

As the role of physician involvement in product development and industry success continues to evolve rapidly in parallel with the evolution of medical devices, orthopedic surgeons have consistently received the greatest average payments compared to other specialties [2,7-14]. Little is known, however, regarding the demographic breakdown of payments within orthopedic surgery by factors such as subspecialty and geographic region [15]. Furthermore, the nature of payment may differ when accounting for payments for consulting, faculty/speaker fees, entertainment/food and beverage, grants, and gifts. As relationships between foot and ankle surgeons and industry continue to burgeon (e.g., orthoses, orthobiologics, and novel implants), we looked to determine how industry payments in this subspecialty changed over time.

The symbiotic relationship that still exists between industry and orthopedic surgeons affords numerous benefits, such as educational opportunities and research funding, and is imperative to the growth of the field [15]. Therefore, the purpose of this study is to investigate and expand on the previously reported trends in payments to foot and ankle-trained orthopedic surgeons between 2014 and 2019. In doing so, as a consequence of the PPSA, the authors examined the (1) median general payments to surgeons, (2) trend in median payments to surgeons across all subcategory payments, (3) trend in median payments to surgeons in 11 regions of the United States, and (4) trend in median payments from the top five companies providing industry payments.

## Materials and methods

A retrospective review of the US CMS OPD was performed to identify all industry payments made by drug and medical device companies to orthopedic foot and ankle surgeons between January 1, 2014, and December 31, 2019 [16]. The OPD is in the public domain and includes all payments/transfers made to licensed physicians across all medical disciplines who receive greater than or equal to $100 annually. Physicians who received total annual payments of less than $100 or no open payments between 2014 and 2019 are excluded from the OPD. Each item is listed in the database with an associated nature of payment: faculty or speaker fees, consulting fees, ownership or investment interest, education, entertainment/food and beverage, gifts, grants, honoraria, royalty or licensing, travel, or lodging [17]. Payments are compiled annually and released as part of datasets. To date, the OPD has released seven datasets beginning in the latter half of 2013. All complete annual datasets from 2014 through 2019 were queried. In concordance with other similar studies [15], the 2013 dataset was excluded due to incomplete data. Given that the OPD is comprised of publicly available data and does not contain protected health information, this study was deemed to be exempt from Institutional Review Board (IRB) review [15].

A total of 28,475 orthopedic surgeons were identified in OPD between the years 2014 and 2019. Compiled data were then stratified by subspecialty to identify all surgeons who were classified as “Allopathic & Osteopathic Physicians/Orthopedic Surgery/Foot and Ankle Surgery,” yielding a sample size of 1,219. All individual industry payments made to orthopedic foot and ankle surgeons were identified and characterized by nature of payment, monetary value in US dollars ($), date of payment, surgeon identifier, the company making payment, recipient State, and US geographic region (i.e., Northeast, Midwest, South, and West) [18,19]. Payments were adjusted to the 2021 Consumer Price Index (CPI) to account for inflation [20]. Companies with multiple listings, as well as those that merged over the six-year period, were compiled into a single entity. The sum total of industry payments was calculated for individual surgeons on an annual basis. Similarly, total payments were stratified by company and nature of payment over time.

Data were analyzed using the Statistical Package for the Social Sciences (SPSS) version 23 (IBM Corp., Armonk, NY). The threshold for statistical significance was set to α < 0.05. Shapiro-Wilk tests of normality were performed on the data, revealing non-normal distributions for all outcome variables of interest (p < 0.001). Consequently, nonparametric statistical tests were performed. Trends in annual payments were assessed via independent-samples Jonckheere-Terpstra tests for a priori ordered alternatives and Spearman’s rank-order correlations. Trends for the number of payments to surgeons per year, payment subtypes, and regional distributions were analyzed similarly. In addition, a pairwise Mann-Whitney U-test with Bonferroni post-hoc correction was performed to compare differences in payments by year. Descriptive statistics and frequencies presented include means, medians, interquartile ranges, percentiles, and sum totals.

## Results

General payments

The study sample consisted of 3,835 foot and ankle surgeons who received 53,280 payments totaling $53,454,850.56 across the six-year time period (see Table 1). Mean and median payments were $1,003.28 and $60.19, respectively. Overall, the vast majority of surgeons (78.4%) received less than $10,000 in payments, accounting for $1,580,321.87 (3.0%) (see Table 2).

**Table 1:** Total number of payments from 2014 to 2019 The Jonckheere-Terpstra test for ordered alternatives demonstrated a significant increase in the median yearly payment with increasing years (p = 0.001). ^a^ US Dollars, adjusted to 2021. ^b^ Pairwise Mann-Whitney U-test compared to 2014, adjusted with Bonferroni corrections.

Year	Sum of Payments^a^ ($)	Payments (N)	Mean Payment^a ^($)	Surgeons (N)	Median Yearly Payment (25th-75th Quartiles)^a^ ($)	p-value^b^
2014	7,438,933.03	7,217	1,030.75	586	56.49 (19.55-178.41)	0.003
2015	6,852,533.17	8,036	852.73	678	50.40 (17.81-160.50)	-
2016	7,545,394.10	8,558	881.68	597	55.21 (17.73-193.30)	0.184
2017	9,460,572.99	9,174	1,031.24	653	71.06 (20.39-229.24)	<0.001
2018	10,124,693.97	9,297	1,089.03	654	77.39 (21.00-277.02)	<0.001
2019	12,032,723.30	10,998	1,094.08	667	56.25 (16.93-197.03)	1
Total	53,454,850.56	53,280	1,003.28	3,835	60.19 (18.78-206.39)	

**Table 2:** Distribution of payments made from 2014 to 2019 Yearly total payment per year including 2014-2019 Open Payments Data. ^a^ US Dollars, adjusted to 2021.

Yearly Total Payment^a^ ($)	Sum of Contributions (%)	Surgeons (N)	Total Surgeons (%)
<100	0	498	12.99
100 to <1000	0.1	539	14.05
1000 to <10,000	2.8	1,375	35.85
10,000 to <100,000	11.6	1,059	27.61
100,000 to <500,000	18.7	233	6.08
≥500,000	66.7	131	3.42

**Figure 1:**
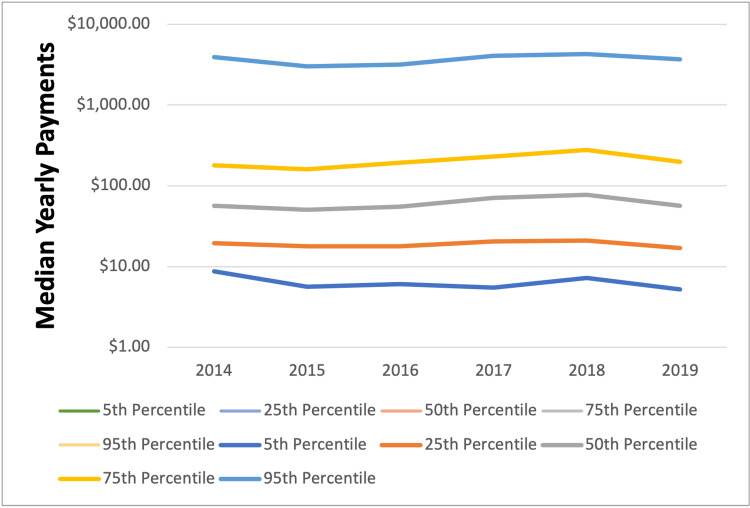
Percentiles across the time of median yearly payments

The top 5% of surgeons (n = 192) received $45,851,924.25, 85.8% of total payments (see Figure 1). Statistically significant differences in mean payment amounts were observed by year (p = 0.001) (see Table 1). Compared to 2015, the index and year with the lowest median payment amount ($50.40), 2014 ($56.49, p = 0.003), 2017 ($71.06, p < 0.001), and 2018 ($77.39, p < 0.001) saw significantly greater individual payment amounts. Additionally, there was a highly statistically significant, strong increase in the number of payments made to foot and ankle surgeons over the six-year period (r = 0.97, p < 0.001). Specifically, the number of payments increased by 52.4% between 2014 and 2019 (n = 7,217; 10,988, respectively). The number of surgeons receiving payments also increased by 13.8% between 2014 and 2019 (n = 586; 667, respectively).

Subcategory payments

With regard to the nature of payments, the greatest increases in median individual payments were observed for gifts and education, which increased by 277.1%, from $26.02 in 2014 to $98.11 in 2019 (r = 0.18, p = 0.05), and by 250.6%, from $264.10 in 2014 to $925.93 in 2019 (r = 0.17, p < 0.001), respectively. There were also significant increases in median payments for royalties and licensing, which increased by 72.1% (r = 0.05, p = 0.04), honoraria which increased by 33.5% (R = 0.23, p = 0.02), and consulting fees which increased by 3.1% (R = 0.042, p = 0.007). There were significant decreases in median payments for faculty or speaker fees, which decreased by 69% from 2,683.92 to 831.36 (R = -0.1, p < 0.001); entertainment, food, and beverage, which decreased by 15.7% from 27.87 to 24.50 (R = -0.01, p < 0.001); and travel and lodging which decreased by 14.3% from 206.17 to 176.66 (R = -0.04, p < 0.001). There were no significant differences in grant payments over time (r = -0.23, p = 0.20).

Regional analysis

Statistically significant increasing trends in median payments over time were observed for the Northeast (p < 0.001) and South regions (p < 0.001); however, the magnitude of these relationships was negligible (r = 0.07, r = 0.03, respectively) (see Tables 3, 4). A very weak, but statistically significant, negative relationship was observed for the Midwest region over time (r = -0.02, p = 0.04). Median payments did not significantly change over time for the Midwest region (r = 0.01, p = 0.35).

**Table 3:** Subtype of payments made from 2014 to 2019 ^a^ US Dollars. IQR: Interquartile range.

	Median (IQR)^a ^($)						p-value	r
	2014	2015	2016	2017	2018	2019		
Faculty or speaker fees	2,683.92 (6,885.93)	895.44 (6,559.14)	1,656.15 (4,686.06)	1,265.71 (3,999.10)	1,582.95 (5,101.56)	831.36 (3,398.18)	<0.001	-0.1
Consulting fees	1,341.96 (3,097.83)	1,119.30 (2,854.21)	772.87 (1,741.72)	1,077.20 (2,100.54)	1,187.21 (2,638.25)	1,383.44 (2,753.88)	0.007	0.042
Education	264.10 (1,037.93)	587.63 (2,212.86)	715.11 (1,779.19)	578.10 (2,188.87)	940.80 (1,112.67)	925.93 (869.55)	<0.001	0.17
Entertainment, food, and beverage	27.87 (61.33)	27.87 (65.91)	26.50 (60.24)	31.13 (78.22)	30.88 (73.42)	24.50 (58.36)	<0.001	-0.01
Gift	26.02 (99.29)		129.73 (121.92)	126.57 (196.62)	25.94 (13.59)	98.11 (173.98)	0.05	0.18
Grant	41,936.25 (22,366.00)	475.71 (8,142.90)	9,884.54 (9,895.49)	10,772.00 (10,098.75)	1,758.84 (7,475.04)	8,841.37 (11,431.20)	0.203	-0.23
Honoraria	805.18 (1,260.88)	899.92 (561.05)	892.11 (764.04)	2,693.00 (4,289.51)	1,840.18 (3,087.81)	1,074.53 (1,743.78)	0.02	0.23
Royalty or license	3,553.12 (1,5870.01)	3,938.64 (15,157.58)	4,707.46 (22,369.81)	5,926.00 (28,043.12)	5,588.96 (17,544.80)	6,115.65 (20,661.54)	0.04	0.05
Travel and lodging	206.17 (344.23)	209.10 (370.72)	233.72 (363.39)	245.69 (332.57)	211.06 (402.54)	176.66 (298.38)	<0.001	-0.04

**Table 4:** Regional data ^a^ US Dollars. IQR: Interquartile range.

Region	Median (IQR)^a^($)						p-value	R
	2014	2015	2016	2017	2018	2019		
Midwest	99.26 (385.77)	83.95 (233.01)	78.88 (307.67)	98.99 (386.14)	88.50 (335.73)	73.24 (293.92)	0.04	-0.02
Northeast	58.24 (227.51)	48.24 (131.04)	53.90 (163.51)	79.37 (219.66)	87.99 (376.25)	81.23 (257.57)	<0.001	0.07
South	34.30 (108.48)	39.16 (122.13)	41.96 (122.98)	48.59 (142.01)	59.74 (190.80)	41.21 (133.98)	<0.001	0.03
West	67.14 (166.49)	55.06 (152.35)	77.30 (218.06)	95.92 (198.63)	85.35 (260.37)	62.52 (147.50)	0.35	0.01

**Figure 2:**
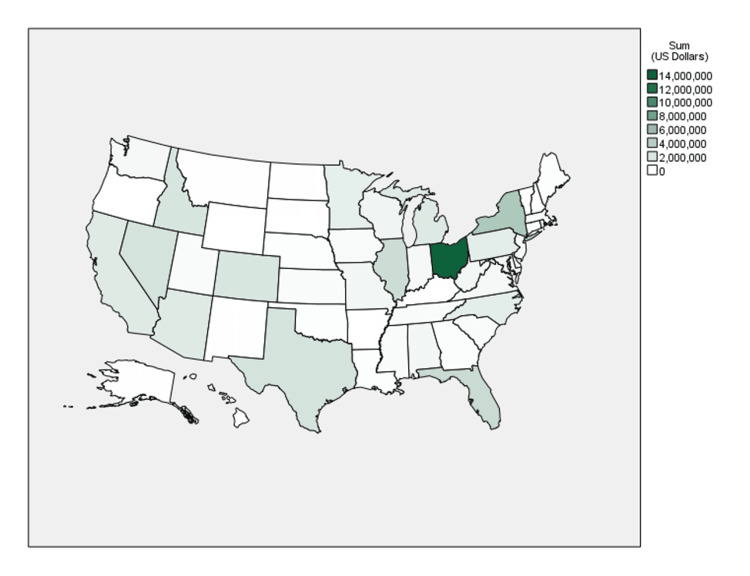
Choropleth map of the sum of payments for all years by state

**Figure 3:**
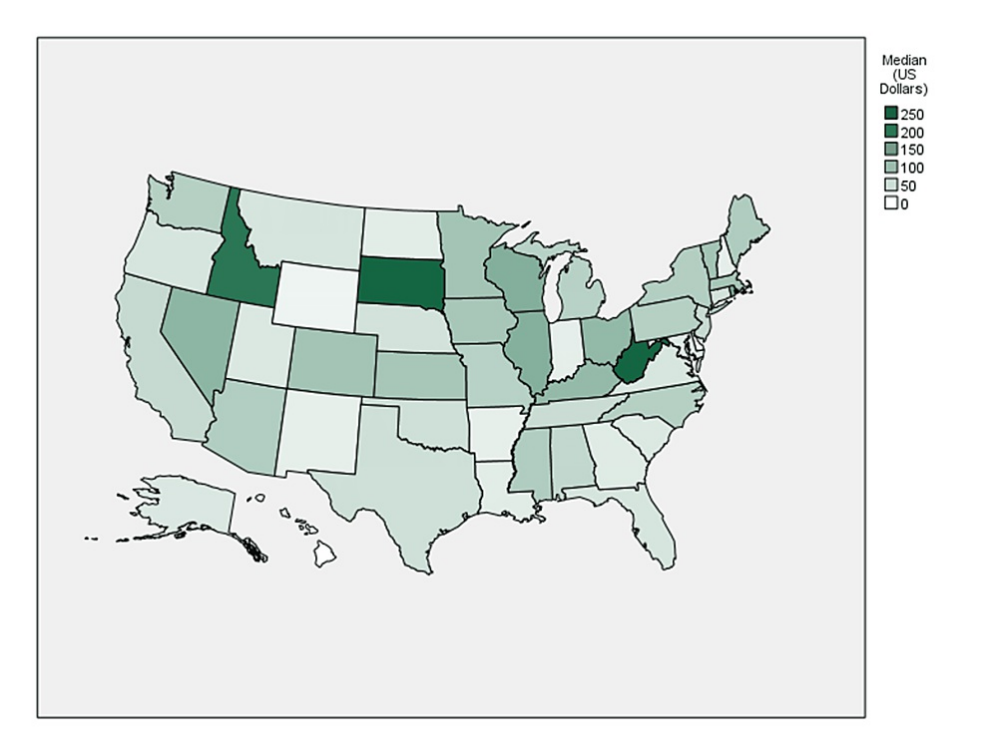
Choropleth map of median yearly total payments per surgeon by state

The Northeast was found to have the largest proportion of sum total payments at 41.8% ($22,320,666.32) as well as the greatest median payment amount ($86.31) (see Figures 2, 3). In contrast, the South had the greatest number of individual payments at 20,157 (37.9%) but the lowest median payment amount ($44.07). Ohio accounted for 24.9% of sum total payments ($13,301,859.78) and 8.2% of the number of individual payments (n = 4,342), the most of any state. West Virginia ($236.32), South Dakota ($227.98), and Idaho ($207.08) saw the largest median payment amounts.

Companies with the highest payments

The top five companies with the highest payments accounted for a total of 75.71% ($40,472,468.86) of all contributions over the six years of the study. The top three contributors made up 67.75% of all payments and included Arthrex, Inc.; Wright Medical Technology, Inc.; and Stryker, Inc., which made up 29.79% ($15,923,387.57,), 29.54% ($15,791,421.25), and 8.42% ($4,500,395.05) of payments, respectively. The fourth and fifth highest contributors were Paragon 28, Inc. and Integra Life Sciences Corp. These companies accounted for 5.48% ($2,931,882.89) and 2.48% ($1,325,382.10), respectively.

**Figure 4:**
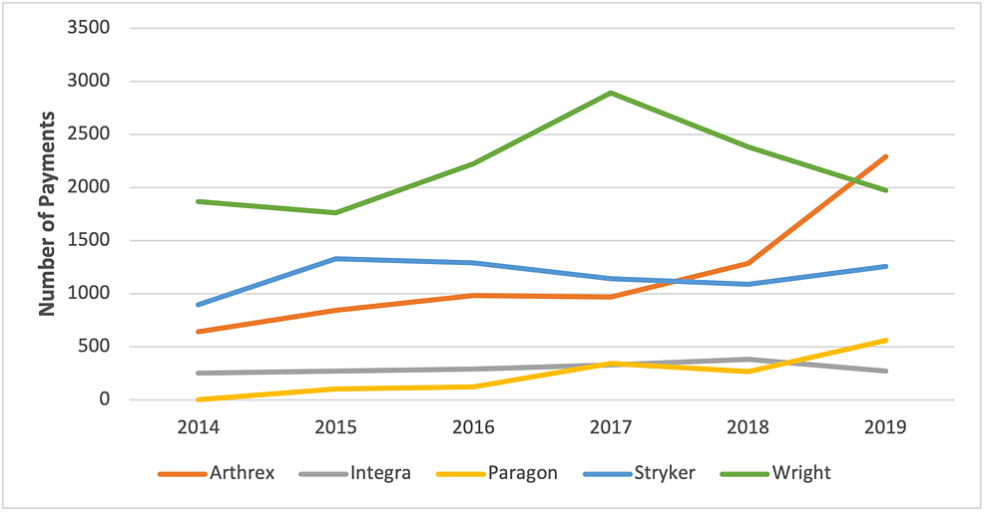
Total number of payments by the top five companies

**Figure 5:**
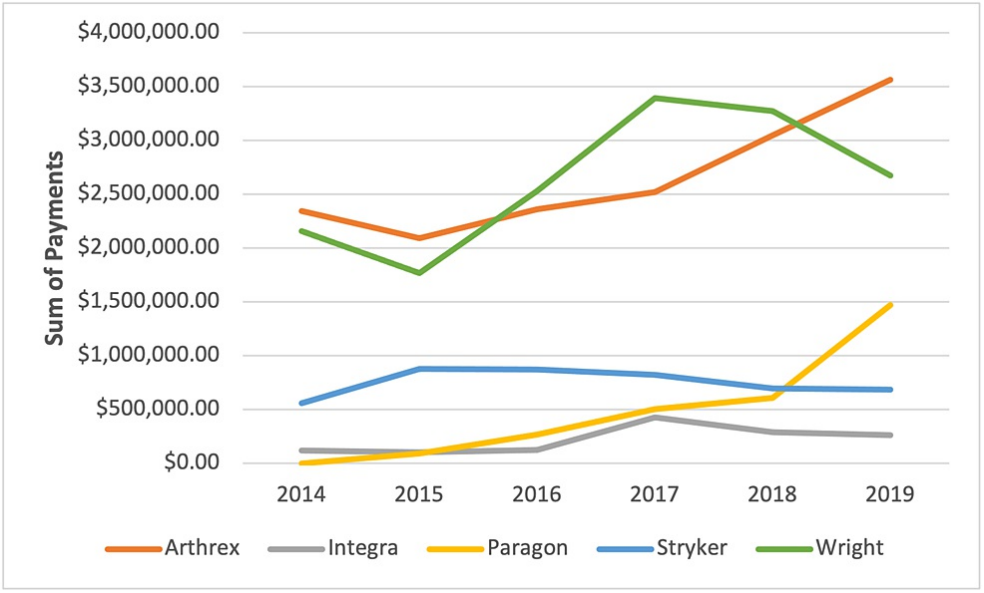
Total sum of payments by the top five companies

The total number of payments made by Arthrex (r = 0.87, p = 0.02) and Paragon (r = 0.93, p = 0.01) increased significantly over the six-year study period (see Figure 4). Similarly, the sum total of payments made by Arthrex (r = 0.89, p = 0.02) and Paragon (r = 0.92, p = 0.01) increased over time (see Figure 5).

**Figure 6:**
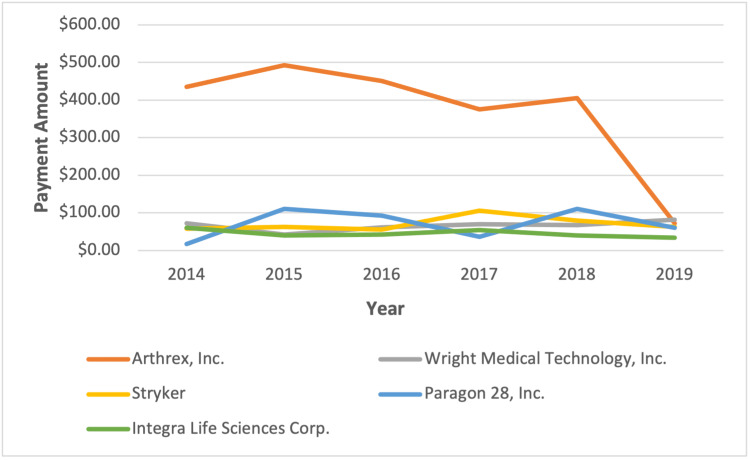
Company median payments to surgeons

Median payments made by Arthrex (r = -0.33, p < 0.001) and Paragon (r = -0.09, p < 0.001) decreased significantly over time, whereas the median payment for Wright increased significantly over time (r = 0.04, p < 0.001) (see Figure 6).

## Discussion

In 2010, the CMS started the National Physician Payment Transparency Program and OPD, which allowed for public access and financial disclosures to be exchanged between physicians and industry. This topic has recently been heavily studied across many orthopedic subspecialties, showing a strengthening of the relationship between the industry and surgeons [13-15]. With the growing innovation within the foot and ankle subspecialty of orthopedics, our goal was to examine if the transparency in industry payments to foot and ankle-trained orthopedic surgeons has changed over six years, from 2014 to 2019. To our knowledge, this is the largest study to detail subcategory industry payments within the foot and ankle subspecialty. Specifically, the authors examined if the transparency in industry payments resulted in the following changes to the (1) median general payments to surgeons, (2) trend in median payments to surgeons across all subcategory payments, (3) trend in median payments to surgeons in 11 regions of the United States, and (4) trend in median payments from the top five companies providing industry payments. Our analysis returned a sample of 3,835 foot and ankle surgeons who received 53,280 payments totaling $53,454,850.56. Over these six years, there was an increase in the number of payments made. The greatest increases were those involving gifts and education, while royalties and licensing also increased. This contrasted with decreases in payments for faculty or speaker fees, entertainment, food and beverage, travel, and lodging. When comparing payment trends per region, the Northeast had the largest proportion of sum total payments as well as the greatest payment amount. The South region also had a significant increasing trend in medium payments, whereas they had the greatest number of individual payments but the lowest median payment amount. Three companies made up 67.75% of all payments across these six years: Arthrex, Inc.; Wright Medical Technology, Inc.; and Stryker, Inc.

A study in 2013 determined that 50.1% of orthopedic surgeons claimed to have a financial relationship with the industry, where they received nearly $80 million within a three-month period [3,21]. In another study that only included foot and ankle surgeons of podiatric medicine, Casciato et al. examined 10,872 payments totaling nearly $70 million among 446 specialists between 2013 and 2020 [22]. The authors determined a higher average value of payment in the later years of the study with a larger percentage of payments being cash and cash-equivalent based. However, our study examined a larger sample size of 3,835 orthopedic foot and ankle surgeons who received 53,280 payments totaling nearly $54 million. Furthermore, our authors identified the breakdown in payment types and the distribution of these payments across the United States while also examining the companies with the most involvement in this subspecialty. Pathak et al. conducted an analysis of the OPD between 2014 and 2017 and determined that 802 orthopedic foot and ankle surgeons received approximately $39 million from the industry through nearly 29,442 transactions [2,21]. Although our study builds off the report by Pathak et al., the authors of our study were able to clearly determine that there were significantly greater individual payment amounts and a significant increase in the number of payments made to foot and ankle surgeons over the six-year period. At the time of their study, Pathak et al. also determined that 91% of the total orthopedic foot and ankle payments were made to the top five percent of orthopedic foot and ankle surgeons [2]. However, the present study determined that this same statistic has most recently dropped to 85.8%, suggesting that there is a greater distribution of these payments across more foot and ankle surgeons. Overall, this analysis provides a renewed understanding of the distribution of payments and how foot and ankle surgeons are more involved with the orthopedic industry.

From 2014 to 2019, there was a shift in payment types, from speaker fees, entertainment, and lodging to education, gifts, honoraria, royalties, and consulting. This increase in payments related to education (250.6%) compares to trends seen in the spine (137%), pediatric (533%), and adult reconstruction subspecialties (1054%) [12,13,15]. As the future of orthopedics continues to grow, the education of orthopedic residents on existing and new hardware, tools, and operative techniques is paramount. Second, we saw royalties and licensing maintain some of the highest dollar amount categories in more recent years. This trend is consistent with previous literature [12,14,15,21]. However, the authors determined that the increase in royalties and licensing occurred in more recent years compared to a previous study examining OPD in adult reconstruction. This could be due to the growing innovation as foot and ankle surgeons are becoming more involved in the industry through the development of medical devices and education.

When examining the geographic distribution of payments, the authors determined that the Northeast maintained the largest sum total and median payment amount compared to other regions, while the state of Ohio accounted for one-fourth of total payments. This differs from a study by White et al., where it was reported that the Midwest region received the greatest sum of payments in their analysis of industry payments within adult reconstruction [15]. A previous OPD study in pediatric orthopedics suggested there may be locality preferences in industry payments based on the location of company headquarters; however, this disagrees with both Pathak et al. and our study [13]. With the increase in virtual conferences and web-based education, it seems that company headquarters location is less of a factor. It is possible that surgeon practice and/or training, regional population, orthopedic volume, or the number of academic institutions affect the change in regional distribution. In either case, further research is needed to evaluate the relationship between regionality and industry payments.

With respect to individual companies, the authors noted that the top three companies made up two-thirds of all payments. Interestingly, while comparing the top companies for adult reconstruction, only one similarity existed - Stryker, Inc. [15]. This difference is likely due to the companies catering their implants to specific subspecialties. Arthrex Inc. has traditionally been used heavily by foot and ankle surgeons. Perhaps, the size or average annual revenue of an individual company plays a role in their industry payments. With that being said, the authors do not believe that the PPSA alone has had a profound effect on industry payments, and it is unclear how the PPSA has affected each company's industry payments.

This study is not without limitations. First, this study is a retrospective review of an existing database. Such databases were not designed to be collected for research, and a great deal of data can be missing. Second, the data is self-reported by industry, and so there is no self-check method to increase the validity of payments or to confirm the accuracy of the data within the database. As such, one could question if the monetary amount or physician specialty is accurate. The OPD was brought about to allow for transparency in the physician/patient industry. However, there is a need for research on patient perception of physician-industry relationships concerning the effect on the orthopedic patient, whether patients are aware of their physician’s relationship with companies, and how physicians can optimize patient care with improved levels of transparency.

## Conclusions

Now that physician-industry financial transactions are readily available to the public, physicians should be wary of the potential implications on their practices and rapport with patients. This transparency can either strengthen the physician-patient connection or create newfound mistrust. In our study, since the OPD became publicly available, we did not identify any significant downward trend in the financial relationship between foot and ankle surgeons and the industry; in fact, we found an increase. This increase in education, royalties, and consulting suggests that more foot and ankle surgeons are getting involved in the industry, which is the opposite of what some might have expected. The partnership between the industry and physicians can help to improve innovation and bring new ideas to the future of orthopedics.
